# Notes on Preparations for the Sick

**Published:** 1907-09

**Authors:** 


### Notes on Preparations for the Sick.

"**Elixoid**" **Formates Compound.**—BURROUGHS, WELLCOME & Co., London.—Each fluid ounce contains:—

Calcium Formate, gr. 12 (0.778 grm.).
Sodium Formate, gr. 6 (0.389 grm.).
Magnesium Formate, gr. 6 (0.389 grm.).

It is claimed that the formates possess marked antiseptic and diuretic properties. "Elixoid" Formates Compound presents a convenient and palatable means of administering the formates of calcium, sodium and magnesium without causing gastric disturbance. Two fluid drachms may be taken thrice daily, in water, after food.

**Quinine Acetyl-Salicylate.**—BURROUGHS, WELLCOME & CO., London.—This is another recent preparation brought out by Messrs. Burroughs and Wellcome, and it is likely to be appreciated by the medical profession.

Acetyl-salicylic acid, or "aspirin," has given satisfactory results in cases where the use of salicylates was deemed necessary, and the combination of this acid with quinine should certainly give results justifying its introduction. No doubt it would be hydrolysed in the duodenum with liberation of quinine and salts of acetic and salicylic acids, and it is thought by some pharmacologists that better results are obtained when active products are thus liberated by hydrolysis within the system, than when they are directly administered.

---

**Tabloids.**—BURROUGHS, WELLCOME & CO., London.—**Gingamint:** soda-mint compound.—Each contains sodium bicarbonate, gr. v.; ammonium bicarbonate, gr. $\frac{1}{2}$; with gingamint, saccharine and oil of peppermint. This preparation is a valuable antacid and stomachic, employed in the relief of dyspepsia, nausea, heartburn and flatulence. It promotes appetite and digestion, relieves griping, and produces a diffusible stimulant effect.

**Slippery Elm.**—Mucilage of slippery elm is largely used as a demulcent and sedative astringent. Alone, or combined with phenol, it is employed locally in pharyngitis and other throat affections, and internally in diarrhœa and dysentery. The mucilage is also stated to have a nutritive value. "Tabloid" Slippery Elm presents a convenient and reliable means of administration. Each represents gr. v. (0.324 gm.) of mucilage of slippery elm, and one may be slowly dissolved in the mouth or swallowed whole with water as required.

**"Tabloid" Carbolic Acid and Slippery Elm.**—Each contains carbolic acid, gr. $\frac{1}{2}$ (0.032 gm.). One may be slowly dissolved in the mouth, or one to two swallowed whole with water twice or thrice daily after food.

---

**Ernutin.**—BURROUGHS, WELLCOME & CO., London.—This drug contains the specific active principles of ergot, the chief of which is the alkaloid ergotoxine. It is physiologically standardised, and presents a uniform degree of activity. For oral administration the dose is 30 to 60 minims. The tabloid for hypodermic use contains gr. $\frac{1}{100}$.

---

**"Wellcome" Brand Anæsthetics.**—BURROUGHS, WELLCOME & CO., London. It is well known that chloroform is subject to decomposition by the action of air and ordinary daylight. To avoid all possibility of deterioration from such influences, "Wellcome" Brand Chloroform is issued in hermetically-sealed amber-coloured glass tubes.

"Wellcome" Brand Ether, S.G. 720, which conforms to the requirements of the British Pharmacopœia for pure ether, is also issued in hermetically-sealed glass tubes. By this method perfectly fresh and chemically pure anæsthetics are always available.

---

**Nutritive Liquid Peptone with Creosote.**—PARKE, DAVIS & Co., London.—This is described as a nutritive liquid, containing peptone and the digested nutritive constituents of malt.

As there are various preparations of peptone on the market, an analysis of the above was made in order to verify the statements concerning it.

We consider it to be one of the best of its kind, especially as it contains nearly 20 per cent. of solid matter, and therefore is more than a simple stimulant. It contains non-coagulable proteid, together with extract of malt. On distilling the liquid, a little alcohol passed over, probably added as a preservative.

It possesses a pleasant flavour, and is altogether an attractive preparation. The combination with creosote and guaiacol (nutritive liquid peptone with creosote) should be very useful and acceptable to patients with chronic lung diseases.

---

**Triple Glycerophosphates with Nuclein** (Chocolate-coated tablets).—PARKE, DAVIS & Co.—These tablets provide the tonic, nutritive and reconstructive properties of the glycerophosphates in association with the bactericidal action of nuclein. Nuclein, it will be remembered, also possesses the valuable power of increasing leucocytosis. This combination is valuable in debilitated conditions generally, and particularly in the various manifestations of tubercular disease, as scrofula, abscess, lupus, adenitis, ulcers and phthisis.

---

**No. 509 Compressed Tablets, Phenol-Phthalein Compound.**—PARKE, DAVIS & Co.—Each of these tablets contains 1 grain of phenol-phthalein, $\frac{1}{500}$ grain strychnine sulphate, and $\frac{1}{100}$ grain of extract of belladonna leaves. The laxative effect of phenol-phthalein is well known; it is reputed to be particularly well adapted for use in habitual constipation, acting without pain or tenesmus. The strychnine and belladonna are valuable auxiliaries, giving tone to the intestinal tract and restoring natural function. The sugar-coated tablet is a particularly acceptable as well as convenient mode of prescribing these drugs. The dose for adults is from three to five tablets at bed-time; for children and delicate women, from one to three tablets will be sufficient.

**Iodalbin.**—PARKE, DAVIS & Co.—Iodalbin is an iodo-proteid compound containing 21 to 25 per cent. of iodine. It is in the form of an almost tasteless powder, insoluble in water, acids or alcohol, but readily soluble in alkaline solutions. When administered, it passes unchanged through the stomach, and is gradually absorbed in the intestine, thus avoiding the gastric irritation that the alkaline iodides are liable to excite, and also ensuring a milder systemic effect. Experiments on animals show the presence of iodine in the saliva very shortly after its administration, but very little can be traced in the fæces. Several months of close clinical observation demonstrate that iodalbin produces the typical alterative effect of the organic iodides without their disadvantages. Iodalbin may therefore be advantageously prescribed in preference to the alkaline iodides in all cases where such treatment is indicated. A smaller dose, 5 to 10 grains, is sufficient, though many patients have taken as much as 60 grains per diem without untoward effects.

---

**Adrenalin and Eucaine Tablets.**—PARKE, DAVIS & Co.—Considerable attention is now being given to local analgesia, as preferable in many cases to total anæsthesia, and for this purpose "Eudrenine" is daily being more and more used. For medical men abroad and for those who prefer to make their own solution at the time of operating, these tablets of Adrenalin and Eucaine have been introduced. Each contains $\frac{1}{2000}$ grain of adrenalin and $\frac{1}{6}$ grain of eucaine lactate, with a proportion of sodium chloride sufficient to impart salinity to the solution. One tablet dissolved in 17 minims of sterile distilled water forms an analgesic and ischæmic agent for use in dental extractions and small operations, containing 1 per cent. of the eucaine salt and about 1 of adrenalin in 30,000 parts. One tablet, dissolved in 85 minims of sterile distilled water forms a solution similar in strength to that used in operations at University College Hospital, as reported in *The Lancet,* July 25th, 1903, and *The British Medical Journal,* December 24th, 1904.

---

**Formidine.**—PARKE, DAVIS & Co.—Formidine is a potent antiseptic suitable for internal or external use. Chemically it is methylene disalicylic acid iodide, a condensation product of iodine, formic aldehyde, and salicylic acid. It is insoluble in water, alcohol and dilute acids. In contact with alkaline organic secretions, it slowly dissolves and develops the characteristic germicidal properties of its constituents, hence it is most useful for bacterial infections of the intestinal tract. The dose is from 1 to 5 grains. Externally it is used in place of iodoform as a stimulating, non-irritating dressing for wounds, ulcers, &c., and as a dusting powder for various skin affections. Its antiseptic power has been proved to be greater than that of iodoform, whilst it is free from offensive odour, does not stain the skin or clothing, does not irritate, and does not produce toxic effects when applied over large areas.

---

**Tannigen** has been found particularly useful *in infantile diarrhœa.* Tannigen is an odourless and tasteless preparation. The fact that it passes unchanged through the stomach, and is not decomposed into tannic acid until it reaches the intestinal canal, renders it of great value in gastro-intestinal affections. Dose: For adults, up to 15 grains; children, 2—5 grains, in milk, as frequently as the occasion may require. A convenient way of administering Tannigen is the tablet form.

---

**Tilia.**—PEEK, FREAN & Co., London.—Tilia is a name given to a milk proteid manufactured by Peek, Frean and Co.

It is a white powder, almost tasteless and odourless, and gives the characteristic proteid reactions when examined chemically.

As a food accessory milk proteids are extremely valuable, being very rich in nitrogen, and readily digested and assimilated.

In addition to Tilia in the form of a powder, Peek, Frean and Co. have put on the market various products containing Tilia, such as cocoa and various kinds of biscuits, to which the introduction of a large percentage of the proteid is guaranteed.

We examined several of these with satisfactory results, but consider the "Diabetic Biscuits" deserving of special notice. They are starch and sugar free, and when placed on a dilute solution of iodine show not the slightest blue colouration—a very delicate test, showing the absence of starch.

We are pleased to speak highly of these preparations.

---

**Pertussin.**—E. TAESCHNER, Berlin.—This preparation is a saccharated elixir, which contains as the chief active ingredient essential oil of thyme. It is recommended for whooping-cough, asthma, catarrh of the larynx, &c. It is not unpleasant to take, and acts as a fairly powerful expectorant.

It is doubtful whether the use of such proprietary articles as this can be justified when so many well-known and approved remedies, prepared from published formulæ, are at the disposal of the physician.

---

**Grape Nuts.**—GRAPE NUTS Co., Shoe Lane, London, E.C.—During recent years grape nuts have been much advertised as a food.

An analysis of the preparation reveals the presence of proteids and soluble carbohydrates (dextrin and reducing sugars), which have been derived from the starch present in the cereals used in the manufacture. Some of the starch has remained unchanged during the preparation of grape nuts; this we consider an advantage for most people.

---

**Claroma: Catarrh Scent.**—J. M. BANNERMAN, Edinburgh.—This inhalant for catarrh is a germicide remedy, a concentrated solution of antiseptics with essential oils. It is free from cocaine and opiates, and it produces a cool, soothing sensation which seems to free the breathing, arrest discharge and sneezing, and give relief to the pain and fulness of the head.

---

**Tablets.**—THE BAYER COMPANY LIMITED, St. Dunstan's Hill, London.

Aspirin.
Helmitol.
Heroin Hydrochl. (gr. $\frac{1}{24}$).
Tannigen.
Trional-Bayer.
Veronal.

Many of the special preparations of this firm are now issued in five-grain tablets. We have received specimens of the above-named. They represent a convenient and handy mode of administration of drugs which are becoming increasingly useful. The tablets are of the highest quality, and they readily disintegrate.

For the chemical analyses mentioned in the above report we have to thank Mr. O. C. M. Davis, B.Sc., A.I.C., of the University College Laboratory.

